# Ethical priority setting for universal health coverage: challenges in deciding upon fair distribution of health services

**DOI:** 10.1186/s12916-016-0624-4

**Published:** 2016-05-11

**Authors:** Ole F. Norheim

**Affiliations:** Department of Global Public Health and Primary Care, University of Bergen, Bergen, Norway; Department of Global Health and Population, Harvard T. H. Chan School of Public Health, Boston, MA USA

**Keywords:** Ethics, Priority setting, Global health, Universal health coverage, Health technology assessment, Health economics

## Abstract

Priority setting is inevitable on the path towards universal health coverage. All countries experience a gap between their population’s health needs and what is economically feasible for governments to provide. Can priority setting ever be fair and ethically acceptable? Fairness requires that unmet health needs be addressed, but in a fair order. Three criteria for priority setting are widely accepted among ethicists: cost-effectiveness, priority to the worse-off, and financial risk protection. Thus, a fair health system will expand coverage for cost-effective services and give extra priority to those benefiting the worse-off, whilst at the same time providing high financial risk protection. It is considered unacceptable to treat people differently according to their gender, race, ethnicity, religion, sexual orientation, social status, or place of residence. Inequalities in health outcomes associated with such personal characteristics are therefore unfair and should be minimized. This commentary also discusses a third group of contested criteria, including rare diseases, small health benefits, age, and personal responsibility for health, subsequently rejecting them. In conclusion, countries need to agree on criteria and establish transparent and fair priority setting processes.

## Background

Worldwide, people now have a reasonable expectation of living long and healthy lives [1]. Avoiding premature mortality is no longer impossible for the majority of people in high-income countries, while the bottom billion still lag behind [2, 3]. In 2015, UN Member States signed Sustainable Development Goal 3: Good health and well-being. The most important sub-target and instrument to reach the remaining targets is universal health coverage (UHC). The Director General of the World Health Organization (WHO) recently said that “*UHC is the ultimate expression of fairness*” and defined it as “*ensuring that everyone can obtain essential health services of high quality without suffering financial hardship*” [4].

This is a radical message – given resource constraints, essential health services cannot entail all possible services but rather a comprehensive range of key services that are well-aligned with other social goals. Priority setting is therefore unavoidable on the path to UHC. Most ethicists even argue that it is unethical to ignore it; indeed, since healthcare needs exceed resource availability, not setting priorities may lead to unfairness. Priority setting ranks services according to their importance and will therefore, by necessity, determine the distribution of services in such a way that it creates winners and losers. How is this done?

WHO and the World Bank have championed cost-effectiveness as a key criterion for global and national priority setting [5, 6]. In the UK, the National Institute for Health and Care Excellence (NICE) identifies the most cost-effective services through health technology assessment, aiming to be open and accountable whilst taking social value judgments into consideration, as recommended by their Citizen’s Council. Priorities are then implemented through clinical practice guidelines and reimbursement rules [7]. In Thailand, the Health Intervention and Technology Assessment Program (HITAP) appraises a wide range of health technologies and public health programs by six criteria: size of the population affected, severity of the disease, effectiveness of health interventions, variation in practice, economic impact on household expenditure, and equity and social implications [8]. In some Latin American countries, including Colombia, Brazil, and Costa Rica, the courts have intervened and ruled that the right to health or healthcare should be the overriding concern [9]. In the US and Germany, comparative effectiveness analysis is widely performed, but cost-effectiveness analysis is not accepted or is seen as unethical [10, 11].

Needless to say, priority setting decisions are controversial in all countries, highlighting the need for clarity and further agreement with regards to priority-setting criteria and processes. The present commentary aims to describe and discuss criteria for fair and ethical priority setting, building on two guidance documents developed by ethicists, economists, health policy experts, and public health and clinical doctors [12, 13]; nevertheless, the views expressed here are my own. The commentary lists three widely accepted cross-cutting criteria and a group of largely unaccepted criteria for priority setting (Table 1), followed by a discussion and rejection of a group of contested criteria. Finally, it argues that countries need transparent processes for priority setting.

**Table 1:** Criteria for priority setting

Accepted criteria	Unacceptable criteria	Contested criteria
Cost-effectiveness	Gender	Size of the population affected (rarity)
Priority to the worse-off	Race	Size of the benefit (very small benefits are ‘irrelevant’)
Financial risk protection	Ethnicity	Age
	Religion	Responsibility for own health
	Sexual orientation	
	Social status	
	Area of residence	

## Discussion

Priority setting occurs at the macro-, meso-, and microlevel of decision making, with a multitude of criteria that could be relevant and have different weights at the various levels.

### Three widely accepted criteria for ethical priority setting

There is agreement among ethical theories that priority setting should be impartial and treat people as equals. There is growing consensus that the aims should be to promote health maximization, fair distribution, and protection against poverty [12, 13]. From these guiding principles three criteria for ethical priority setting arise, namely (1) cost-effectiveness, (2) priority to the worse-off, and (3) financial risk protection.

Choosing priority services based on the cost-effectiveness of new interventions compared to current standard is important considering that improving the length and quality of life has both direct and indirect value for people. Even if cost-effectiveness is not accepted in Germany and the US, there are very few ethicists who would argue that this criterion is not at all relevant – to not improve health as much as possible would have substantial opportunity costs in terms of healthy life years forgone [14]. In addition, the most cost-effective services often benefit the worse-off and provide the most financial risk protection, although this is not always the case. However, cost-effectiveness cannot be the only criterion.

Priority to the worse-off is important since benefiting them matters more than those who are better-off and would reduce unfair inequalities [15]. The worse-off can be defined as those with the most severe and large individual disease burden, or the poorest or otherwise disadvantaged [13]. Since the most cost-effective services do not always benefit the worse-off, services targeting the worse-off should be assigned extra value.

Financial risk protection is important, especially in low-income settings, since disease may cause substantial loss of income or because out-of-pocket expenditure for health services may impoverish people [16]. In cases where less cost-effective services may provide very high financial protection, such services should be given extra priority.

### Unacceptable criteria

There is agreement among ethical theories about unacceptable criteria for priority setting. Even if these are widely used in practice, it is not considered acceptable to treat people differently according to their gender, race, ethnicity, religion, sexual orientation, social status, or area of residence [12, 13]. These criteria are ethically unacceptable since they are morally irrelevant and priority setting should only take relevant criteria into consideration; health outcome inequalities associated with such personal characteristics are therefore unfair and should be minimized.

### Contested criteria

There is a long list of additional criteria where there is less consensus and where countries may vary with regards to the social value judgments they make.

The size of the population affected is an often proposed criterion, at times assigning higher priority to rare diseases and at others to common diseases (large aggregate disease burden). My response is that prevalence is not ethically relevant. Since it is hard to develop treatments for rare diseases, many countries (rightly so) require less strict documentation of evidence for effectiveness. However, the ethical importance of services for rare and very common diseases is captured by the three accepted criteria discussed above.

Some have argued that very small benefits for non-severe conditions are morally “irrelevant” and should not be aggregated and compared to large benefits for few people [17]. My response is that all health benefits are relevant. If curing a headache is morally irrelevant, it would never get priority. Cost-effectiveness balanced against priority for the worse-off would, for all practical purposes, capture what is ethically relevant.

Age is sometimes used in prioritizing between patients for scarce organ transplants, but is rarely recommended at the group level as a criterion in itself. Some argue that the indirect effect of using cost-effectiveness and priority to the worse-off is age discriminatory. My response is that there are no age-neutral principles for priority setting in health since a long lifespan is inherent in the definition of health. If we aim to improve healthy life expectancy and reduce inequality in longevity, the young will typically benefit first. This is also ethically acceptable [18]; however, age itself should not be a criterion.

Some argue that patients who are responsible for their poor health status due to their freely chosen lifestyle should be given lower priority than others. My response is that society’s primary responsibility is to enable equal opportunities through health service provision and fair distribution of the determinants of health. The expected external cost of choice can be covered through taxes (e.g., tobacco) without making people responsible for the outcome of their choices [19].

Finally, comorbidity, the occurrence of two or more conditions at the same time, is often seen as a challenge for priority setting at the clinical level. Should the presence of dementia count when assessing priority for patients in need of advanced chemotherapy for cancer? Should metastatic cancer count when assessing priority for patients in need of a heart transplant? Comorbidity may reduce the overall effect and value of such treatment. My response is that all medically relevant facts should be carefully considered and that comorbidity may make a patient worse-off than others and count in that patient’s favor, but must be balanced against the reduced effect and cost-effectiveness of the service; indeed, a heart transplant would be wasted if the patient is likely to die of terminal cancer a month later.

### The role of fair processes

Some argue that all substantive criteria for priority setting are so contested that they should be replaced by a fair and legitimate process. My response is that both process and substantive judgments are important. “Accountability for Reasonableness” is a widely accepted framework that sets out conditions for a legitimate priority setting process [20]. The core idea is that governments or other providers should make explicit the range of services they offer, and that reasons for inclusion or exclusion are made transparent to all affected parties. A fair process is inclusive and has broad stakeholder involvement and mechanisms for critical assessment and revision. The process itself should be institutionalized. If satisfied, these conditions can connect decisions about priority setting to broader democratic processes [18].

### Future directions and conclusions

Priority setting is inevitable on the path towards UHC and better population health. There is growing consensus regarding the three key criteria for ethical priority setting, namely cost-effectiveness, priority to the worse-off, and financial risk protection. Herein, it is argued that other, contested criteria are unnecessary or unacceptable, but that countries need transparent, legitimate processes for priority setting. A suitable starting point would be to establish a global priority setting commission, as well as commissions at the national level, to develop guidelines for efficient and fair priority setting.
